# Effects of pre-exercise acupuncture stimulation on heart rate response during short-duration exercise

**DOI:** 10.1186/s13102-021-00358-1

**Published:** 2021-10-16

**Authors:** Hidehiro Nakahara, Shin-ya Ueda, Eriko Kawai, Rui Higashiura, Tadayoshi Miyamoto

**Affiliations:** 1 Graduate School of Health Sciences, Morinomiya University of Medical Sciences, 1-26-16 Nankokita, Suminoe, Osaka City, Osaka 559-8611 Japan; 2 Faculty of Education, Gifu University, 1-1 Yanagido, Gifu, 501-1193 Japan; 3 RIKEN Center for Biosystems Dynamics Research Laboratory for, Pathophysiological and Health Science, 6-7-3 Minatojima-minamimachi, Chuo-ku, Kobe, Hyogo 650-0047 Japan; 4 Osaka Kyoiku University Graduate School of Education, 4-698-1 Asahigaoka Kashiwara, Osaka, 582-8582 Japan; 5 Graduate School of Human Environment Faculty of Sport and Health Sciences, Osaka Sangyo University, Wellness 2008, 3-1-1, Nakagaito, Daito, Osaka 573-1004 Japan

**Keywords:** Acupuncture effects, Bradycardia, Low-intensity exercise, High-intensity exercise

## Abstract

**Background:**

The purpose of the present study was to investigate the effects of bradycardia induced by pre-exercise acupuncture on heart rate responses during short-duration exercise.

**Methods:**

A total of 29 healthy subjects underwent two protocols: protocol 1 assessed the effects of manual acupuncture on heart rate response during rest, and protocol 2 tested the hypothesis that the bradycardic effects induced by pre-exercise acupuncture continue during low- and high-intensity exercise. Their average age, height, weight, and body mass index were 21.2 ± 2.0 years, 167.2 ± 8.8 cm, 63.8 ± 12.8 kg, and 22.7 ± 3.5 kg/m^2^, respectively. In acupuncture stimulations for protocols 1 and 2, an acupuncture needle was inserted into the lower leg and manual acupuncture stimulation was performed at 1 Hz.

**Results:**

In protocol 1 (resting condition), acupuncture stimulation induced a bradycardic response, which continued for 4 min after the cessation of acupuncture stimulation (p < 0.05). In protocol 2, the bradycardic response induced by pre-exercise acupuncture stimulation remained during low-intensity exercise and in the beginning of high-intensity exercise performed immediately after the cessation of acupuncture stimulation (p < 0.05). However, the effects disappeared when post-acupuncture exercise was performed when the heart rate was approximately 140 beats/min during high-intensity exercise. The rating of perceived exertion after exercise differed significantly between the acupuncture stimulation task (7.9 ± 1.6) and no-stimulation task (8.5 ± 2.0) (p = 0.03) only in the low intensity group.

**Conclusion:**

This study may provide new insights into the effect of acupuncture stimulation on psycho-physiological conditions during exercise.

## Introduction

Acupuncture has been used to affect cardiovascular regulatory function through modulation of the autonomic nervous system. Our study in humans suggested that electroacupuncture at the Ximen acupuncture point (acupoint) (WHO; PC4) elicits significant depressor and bradycardic responses [1]. In addition, in our recent study, acupuncture stimulation at the lower leg, ear, abdomen, and forearm elicited different blood pressure and heart rate responses in humans [2]. In particular, a significant depressor response was observed only by lower leg stimulation, whereas significant bradycardic responses were observed by stimulation at all target acupoints. Nishijo et al. [3] also reported that manual acupuncture stimulation at the PC4 acupoint for 30–60 s induced a bradycardic response.

An animal study by Michikami et al. [4] demonstrated that electroacupuncture stimulation reduces arterial blood pressure and sympathetic nerve activity. Electroacupuncture stimulation shifted the sympathetic nerve activity‒intracarotid sinus pressure relationship (neural arc) to lower sympathetic nerve activity, suggesting that electroacupuncture reduces sympathetic nerve activity by resetting the arterial baroreflex neural arc. In humans, Nishijo et al. [3] also proposed that the bradycardic response induced by acupuncture stimulation is mediated by both an increase in cardiac vagal activity and a decrease in sympathetic nerve activity because the response was attenuated by sequential autonomic blockade with atropine and propranolol.

Acupuncture has also been regarded as an effective method to improve exercise performance. Indeed, Gentil et al. [5] reported that subjects who underwent acupuncture and moxibustion sessions twice a week for a period of 5 weeks had a lower heart rate associated with an increased velocity in the anaerobic threshold during treadmill exercise after the intervention period. They suggested that the improvement of exercise performance is associated with decrease in the heart rate resulting from modulation of the autonomic nervous system by acupuncture treatment. Lin et al. [6] reported that acupuncture beginning 15 min prior to exercise induced the rapid recovery of heart rate and blood lactic acid after exercise. White and Raven [7] reported that the increase in heart rate elicited by dynamic exercise depended on the balance between the influences of the parasympathetic and sympathetic branches of the autonomic nervous system. These studies suggest that modulation of the autonomic nervous system elicited by pre-exercise acupuncture stimulation continues during exercise that activates sympathetic vasoconstrictor outflow and tachycardia. In addition, the reduction in heart rate elicited by acupuncture stimulation may play an important role in regulating myocardial oxygen utilization during exercise, especially submaximal exercise. Indeed, a previous study found that the heart rate is correlated with myocardial oxygen consumption [8, 9]. Therefore, it can be reasonably assumed that the reduction in heart rate elicited by acupuncture stimulation will improve cardiac energy efficiency during exercise. However, it remains unclear whether acupuncture stimulation immediately before exercise induces cardiovascular benefits during exercise and whether the effects depend on exercise intensity.

Based on previous studies, we hypothesized that acupuncture administered before exercise alters the autonomic nervous system and these effects continue during exercise performed after cessation of acupuncture. Therefore, the present study investigated the effects of bradycardia induced by pre-exercise acupuncture on heart rate responses during low- and high-intensity exercise. This study may provide new insights into the effects of acupuncture stimulation on physiological conditions in athletes and/or during exercise.

## Methods

### Subjects

Twenty-nine healthy subjects [16 men and 13 women; age, 21.2 ± 2.0 (mean ± SD) years; height, 167.2 ± 8.8 cm; weight, 63.8 ± 12.8 kg; body mass index, 22.7 ± 3.5 kg/m^2^] participated in the present study. Participants were recruited with the following inclusion criteria: aged between 18 and 29 years old; and no cardiovascular and pulmonary disorders. The subjects were healthy sedentary subjects (n = 12), recreational soccer players (n = 4), recreational runners (n = 2), recreational baseball players (n = 2), recreational basketball players (n = 6), and recreational Judoists. (n = 3). Written informed consent was received from all subjects after they were given full explanations of the objectives, methods, and potential risks of the study. All protocols were reviewed and approved by The Ethics Committee of Morinomiya University of Medical Sciences (No. 2019-135) and were conducted according to the Declaration of Helsinki.

Height and weight were measured as standard practice on the first day of the experiment. The body mass index (BMI) was calculated as the ratio of weight to height squared (kg/m^2^).

### Protocol 1 (n = 8)

#### Subjects and experimental protocols

The subjects had a mean (± SD) age of 22.9 ± 2.3 years, mean height of 165.8 ± 6.6 cm, mean weight of 60.9 ± 14.1 kg, and mean BMI of 21.9 ± 4.2 kg/m^2^. This experiment was designed to observe the effects of acupuncture stimulation on heart rate response under resting conditions, comprising the effects during 10 min of stimulation and continuation of the effects for 10 min after the cessation of stimulation.

The subjects were requested to remain calm in a resting supine position for approximately 10 min to achieve a stable cardiovascular status before the experiment. Then, disposable electrocardiography (ECG) electrodes were attached to the chest to monitor the heart rate by a three-lead ECG telemeter (BSM-7201, Nihon Kohden Co., Tokyo, Japan). The heart rate was recorded for the last 2 min of the resting period (pre-stimulation). Each subject then received acupuncture stimulation for 10 min on the right side. A stainless-steel acupuncture needle (diameter, 0.16 mm; length, 40 mm; CE0123, Seirin) was inserted into the Zusanli acupoint (WHO; ST 36), which overlies the deep peroneal nerve and tibialis anterior muscle, to a depth of 5 mm.

#### Acupuncture intervention

Acupuncture stimulation at an acupoint causes a reflex-induced bradycardic response [2]. A previous study revealed that simulation of two sets of acupoints simultaneously does not cause additive cardiovascular responses [10]. Therefore, only the Zusanli acupoint on the right side was stimulated. The acupuncture needle was moved up and down by approximately 5 mm at a frequency of 1 Hz during acupuncture stimulation [2]. The acupuncture needle was removed immediately after 10 min of stimulation. Thereafter, each subject was again requested to remain calm in a resting supine position for 10 min (recovery period).

### Protocol 2 (n = 21)

#### Subjects

**Fig. 1:**
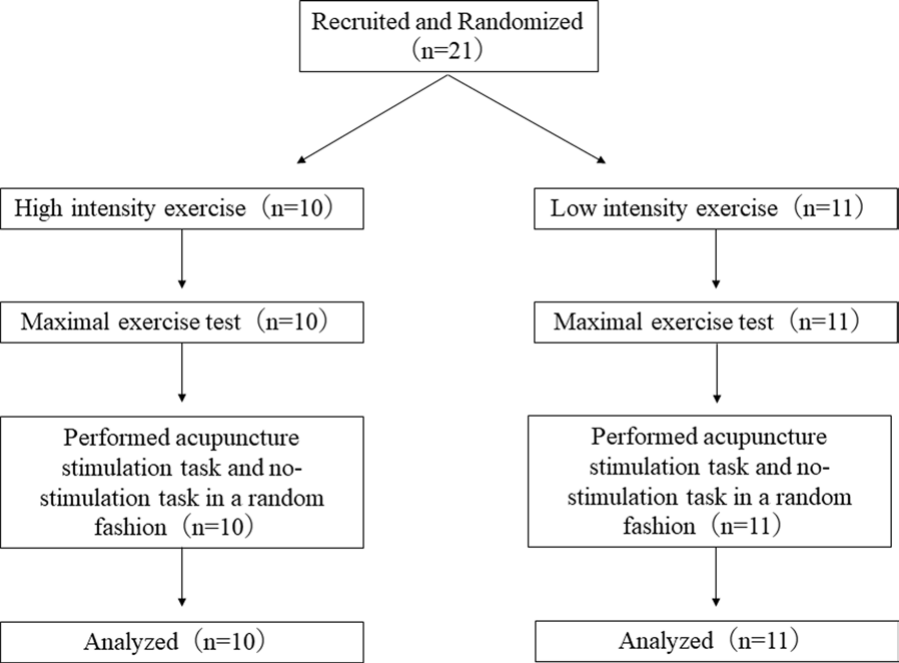
Flow chart of the study design in protocol 2

This experiment was designed to observe the effects of acupuncture-induced bradycardia on heart rate response during subsequent low- and high-intensity exercise. The effects of pre-exercise acupuncture stimulation was assessed in a randomized crossover design (see Fig. 1). Subjects were randomly assigned to one of two groups by a lottery: high intensity group (n = 10) and low intensity group (n = 11). All subjects were assessed on 3 separate days. One week before scheduled data collection, all subjects came to the experimental room in the laboratory to perform the maximal exercise test.

### Maximal exercise test

The subjects underwent a maximal exercise test. A computer-controlled bicycle ergometer (232CXL; Combi Co., Tokyo, Japan) with an incremental protocol was used to assess the maximal work rate. The work rate was set at 20 W initially and increased by 1 W every 3 s until the subject was no longer able to maintain a pedaling frequency of 60 rpm despite strong verbal encouragement.

## Experimental protocols

After arriving at the laboratory on the day of data collection, each subject wore a Polar chest strap and Polar heart rate monitor (V800; Polar, Kempele, Finland) to measure the heart rate, and was requested to remain calm in a resting supine position for approximately 10 min. Then, the heart rate was recorded during the last 2 min of the pre-experimental period (baseline value). Perceived exertion (RPE, scale 6–20) was also rated immediately after all high- and low-intensity exercise tasks.

### High-intensity exercise

In the high intensity group, each subject performed two tasks after the baseline heart rate was measured: (task 1) acupuncture stimulation under resting conditions in the supine position for 10 min, and upon cessation of acupuncture, perform high-intensity exercise at 100% of maximal work (acupuncture stimulation task); (task 2) rest in supine position for 10 min without undergoing acupuncture stimulation and then perform high-intensity exercise as in task 1 (no-stimulation task). Exercises were conducted using a bicycle ergometer at a constant load of 100%. The pedaling speed was also maintained between 55 and 60 rpm. Each subject performed the exercise for 1 min. The study was conducted to examine the influence of pre-exercise acupuncture on short-duration, anaerobic exercise. A duration of 1 min was considered sufficient to assess anaerobic capacity, to avoid excessive contribution from the aerobic energy pathways, and ensured the completion of the test [11]. Subjects were also randomly assigned to one of two tasks by a lottery: task 1 and task 2. The two tasks were tested on separate days with a 7-day interval (washout period) [12].

### Low-intensity exercise

Each subject in the low intensity group performed two tasks after measuring the baseline heart rate: (task 1) acupuncture stimulation under resting conditions in the supine position for 10 min, followed by low-intensity exercise at 20% of maximal work (acupuncture stimulation task); (task 2) rest in the supine position for 10 min without undergoing acupuncture stimulation, followed by low-intensity exercise as in task 1 (no-stimulation task). Exercises were conducted using a bicycle ergometer at a constant load of 20% of the maximum work rate. Exercise protocols were the same as for high-intensity exercise.

### Acupuncture intervention

In the acupuncture stimulation task, a stainless-steel acupuncture needle (diameter, 0.16 mm; length, 40 mm; CE0123, Seirin) was inserted into the Zusanli acupoint (WHO; ST 36). The acupuncture stimulation was performed as in protocol 1.

### Data analysis

#### Protocol 1

The heart rate was sampled at 200 Hz and stored on a laboratory computer system throughout the entire experimental period. The heart rate value was averaged in 2-min segments as follows: last 2 min of pre-stimulation period (baseline); 0–2, 2–4, 4–6, 6–8, and 8–10 min of the acupuncture stimulation period; 0–2, 2–4, 4–6, 6–8, and 8–10 min of the recovery period.

#### Protocol 2

In protocol 2, the heart rate values were averaged as follows: last 2 min of baseline condition (baseline); 0–10 min of resting condition with or without acupuncture stimulation (resting); 20-s segments (0–20, 20–40, 40–60 s) during low- or high-intensity exercise for 1 min (exercise).

### Statistical analysis

In protocol 1, differences in heart rate among the 11 segments (baseline; 0–2, 2–4, 4–6, 6–8, and 8–10 min of the acupuncture stimulation period; 0–2, 2–4, 4–6, 6–8, and 8–10 min of the recovery period) were examined using a repeated-measures one-way analysis of variance (ANOVA) with Dunnett's post hoc test. In protocol 2, the baseline heart rate and RPE after exercise were compared between the acupuncture stimulation task and no acupuncture stimulation task using the paired Student’s t-test. Changes in heart rate relative to the baseline value at different periods during the task were compared between acupuncture stimulation and no acupuncture stimulation using two-way analysis of variance (ANOVA). Independent variables were stimulation (with or without acupuncture stimulation) and condition (resting condition; 0–20, 20–40, and 40–60 s of exercise condition). A *p*-value less than 0.05 was considered significant.

## Results

### Protocol 1

**Fig. 2:**
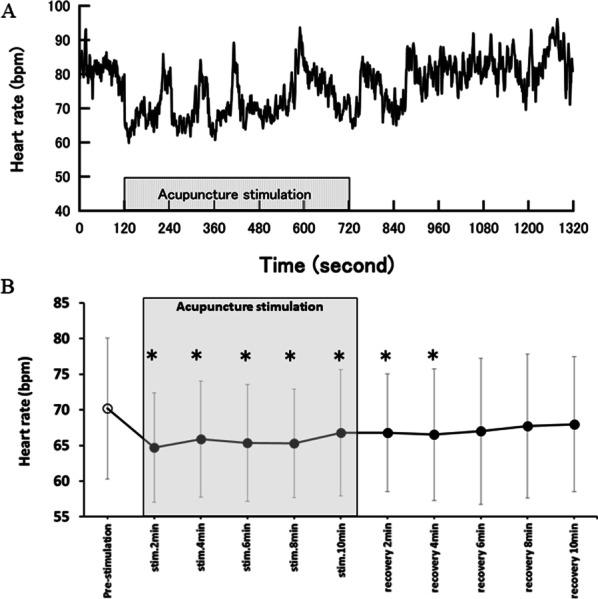
Representative traces of heart rate responses obtained in one subject (**A**) and heart rate averaged during pre-stimulation, acupuncture stimulation, and recovery periods for all subjects (**B**) in protocol 1. *p < 0.05: significantly different from pre-stimulation period

Representative traces of the heart rate response recorded in one subject are shown in Fig. 2A. The heart rate decreased immediately after acupuncture was started and the decrease persisted during the acupuncture stimulation period. After acupuncture stimulation, the heart rate returned to the pre-stimulation level gradually during the recovery period.

Heart rates averaged for all subjects during the pre-stimulation period, acupuncture stimulation period, and recovery period are shown in Fig. 2B. ANOVA revealed a significant main effect [*F*(10, 70) = 3.16, p = 0.02]. Post hoc test revealed significant decreases in heart rate during the entire acupuncture stimulation period and for the first 4 min of the recovery period compared with the pre-stimulation period. However, no significant differences were observed for the last 6 min of the recovery period compared with the pre-stimulation period.

### Protocol 2

Subject characteristics and maximal work rate during the incremental exercise protocol are shown in Table 1. Heart rate responses in each condition during the no-stimulation task and acupuncture task are shown in Table 2. In the high intensity group, there was no significant difference in baseline resting heart rate between the no-stimulation task and acupuncture stimulation task (54.3 ± 6.5 beats/min and 53.0 ± 7.3 beats/min, respectively). In the low intensity group, there was also no significant difference in baseline resting heart rate between the no-stimulation task and acupuncture stimulation task (53.8 ± 5.0 beats/min and 55.2 ± 5.5 beats/min, respectively).

**Table 1:** Physiological characteristics for subjects in protocol 2

	Hight intensity group (n = 10)	Low intensity group (n = 11)
Age (year)	20.5 ± 1.6	20.7 ± 1.5
Height (cm)	168.0 ± 8.8	167.9 ± 9.1
Weight (kg)	66.4 ± 12.5	66.4 ± 12.9
Maximal work load (Watt)	274.9 ± 53.2	274.9 ± 54.9

Data are expressed as mean ± standard deviation (SD)

**Table 2:** Heart rate responses during each condition both no-stimulation task and acupuncture stimulation task

Group	Variable	No stimulation task				Acupuncture stimulation task			
		Resting value	Exercise			Resting value	Exercise		
			0–20 s	20–40 s	40–60 s		0–20 s	20–40 s	40–60 s
High intensity group	Heart rate (beats/min)	55.6 ± 4.9	100.1 ± 10.1	130.0 ± 10.4	145.1 ± 6.6	51.6 ± 7.5	94.0 ± 11.2	124.0 ± 9.9	142.0 ± 7.4
Low intensity group	Heart rate (beats/min)	55.5 ± 4.3	81.0 ± 6.6	90.0 ± 6.1	97.5 ± 6.0	52.8 ± 4.1	80.0 ± 5.0	87.0 ± 5.6	94.8 ± 4.0

Data are expressed as mean ± standard deviation (SD)

**Fig. 3:**
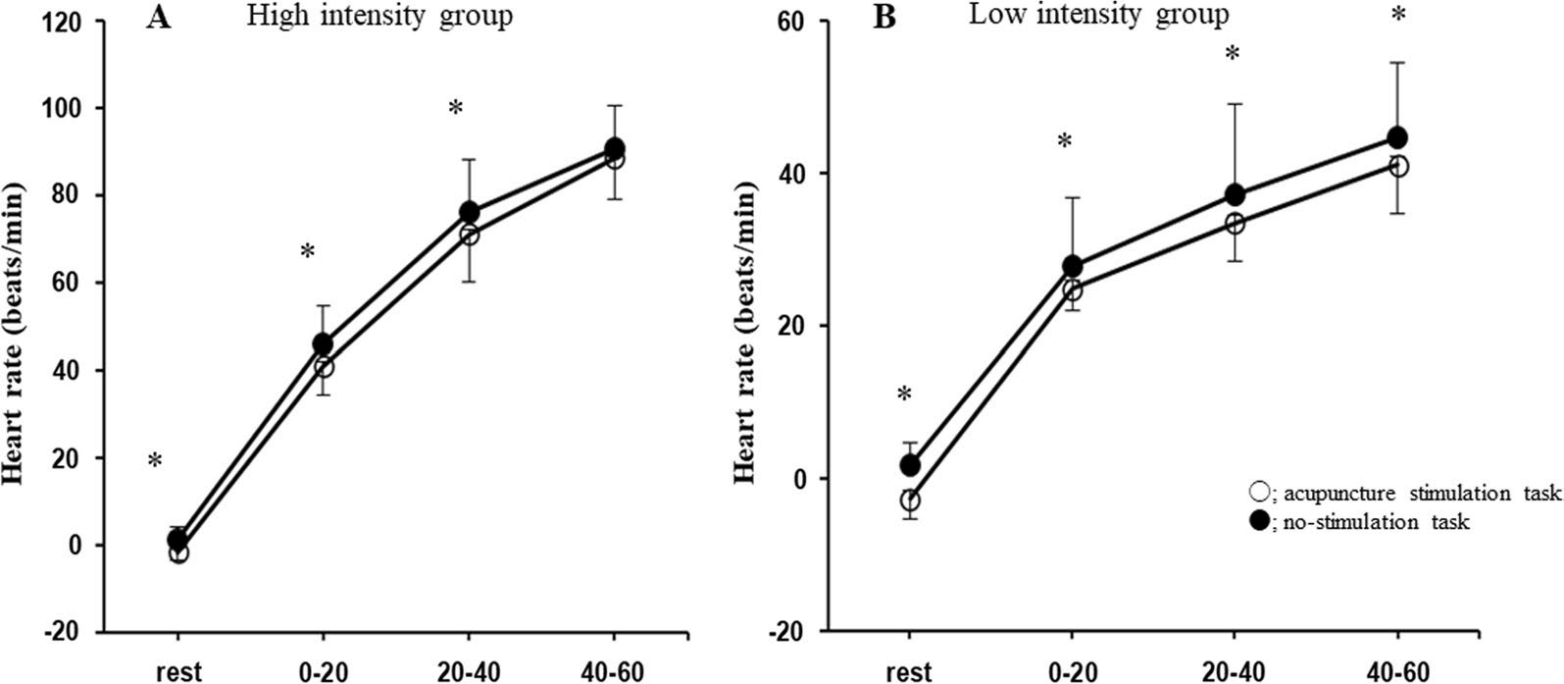
The mean heart rate for all subjects during rest, and 0–20, 20–40, and 40–60 s of high- (**A**) or low-intensity exercise (**B**) during two experimental tasks. All data represent changes from baseline resting values. In the high-intensity exercise condition, ANOVA revealed a significant main effect of stimulation (F(1, 9) = 14.3, p = 0.004) and condition (F(3, 27) = 436.2, p < 0.001) on heart rate. However, there were no significant stimulation x condition interaction effects. In the low-intensity exercise condition, ANOVA revealed a significant main effect of stimulation (F(1, 10) = 13.9, p = 0.004) and condition (F(3, 30) = 745.2, p < 0.001) on heart rate. However, there were no significant stimulation x condition interaction effects. Data are the mean ± standard deviation. *p < 0.05 significant difference between acupuncture stimulation and no-stimulation tasks

The mean changes in heart rate relative to the baseline value of all subjects, in the 10-min resting condition (with or without acupuncture), and at 0–20, 20–40, and 40–60 s during high-intensity exercise for the acupuncture stimulation and no-stimulation tasks are presented in Fig. 3A. ANOVA revealed a significant main effect of stimulation [*F*(1, 9) = 14.3, p = 0.004] and condition [*F*(3, 27) = 436.2, p < 0.001] for heart rate. However, there was no significant interaction between stimulation and condition. Post hoc test demonstrated significantly smaller changes in heart rate during the acupuncture stimulation task than during the no-stimulation task in the 10-min resting condition (p = 0.02), and at 0–20 (p = 0.02) and 20–40 s (p = 0.04) of exercise. However, the change in heart rate at 40–60 s of exercise was not significantly different between the acupuncture stimulation task and no-stimulation task.

In the high intensity group, the RPE after exercise did not differ significantly between the acupuncture stimulation task (15.4 ± 2.0) and the no-stimulation task (15.2 ± 1.8).

The mean changes in heart rate relative to baseline value of all subjects, during the 10-min resting condition (with or without acupuncture), and at 0–20, 20–40, and 40–60 s during low-intensity exercise for the acupuncture stimulation and no-stimulation tasks are depicted in Fig. 3B. ANOVA revealed a significant main effect of stimulation [*F*(1, 10) = 13.9, p = 0.004] and condition [*F*(3, 30) = 745.2, p < 0.001] on heart rate. However, there was no significant interaction effect between stimulation and condition. Post hoc test revealed significantly lower changes in heart rate with the acupuncture stimulation task than with the no-stimulation task during the resting period (p < 0.001), and at 0–20 (p < 0.05), 20–40 (p = 0.03), and 40–60 s (p < 0.05) of exercise.

In the low intensity group, the RPE after exercise differed significantly between the acupuncture stimulation task (7.9 ± 1.6) and the no-stimulation task (8.5 ± 2.0) (p = 0.03).

## Discussion

The present study observed significant bradycardic responses during acupuncture stimulation in the lower leg and continuation of the effects for 4 min after the cessation of acupuncture stimulation. Furthermore, the acupuncture-induced bradycardic effects remained during 1-min low-intensity exercise and in the beginning of 1-min high-intensity exercise performed after the cessation of acupuncture. However, the effects were masked when the heart rate increased to approximately 140 beats/min during high-intensity exercise (Table 2).

The animal study conducted by Uchida et al. [13] found that the heart rate decreased significantly within 20–40 s after the start of acupuncture stimulation in the hindlimb for 1 min and that acupuncture-induced bradycardia continued for 20–40 s after acupuncture stimulation was terminated. Yamamoto et al. [14] demonstrated that manual acupuncture stimulation elicited decreases in arterial pressure and heart rate in rats, and that the minimum arterial pressure and heart rate were reached near the end of the acupuncture stimulation period. They also reported that manual acupuncture stimulation reduced the heart rate, which gradually returned to the baseline value after the cessation of acupuncture stimulation.

In a human study, Nishijo et al. [3] proposed that the acupuncture-elicited bradycardic response is mediated by both an increase in cardiac vagal activity and a decrease in sympathetic nerve activity because the response was attenuated by sequential autonomic blockade with atropine and propranolol. Michikami et al. [4] reported that acupuncture stimulation at ST36 resets the arterial baroreflex neural arc to reduce sympathetic nerve activity. In addition the reflex pathway of the acupuncture-elicited heart rate decrease involves mainly group IV muscle afferent fiber activity, which leads to the activation of GABA receptors in the brainstem [13, 15].

The bradycardic effects induced by pre-exercise acupuncture remained during low-intensity exercise and the beginning of high-intensity exercise performed immediately after the cessation of acupuncture stimulation. Previous studies suggested that acupuncture improves exercise performance and expedites post-exercise recovery [5, 6]. Cheung and Jones [16] reported that transcutaneous electrical nerve acupuncture stimulation at the PC6 acupoint both before and immediately after exercise facilitates heart rate recovery after high-intensity exercise. Furthermore, the long-term effects of acupuncture on cardiovascular responses during exercise have been reported. Lin et al. [6] demonstrated that acupuncture stimulation at PC6 and ST36 acupoints 15 min prior to exercise induces the rapid recovery of heart rate and blood lactic acid after exercise. Gentil et al. [5] proposed that an acupuncture session twice a week for 5 weeks reduces the heart rate response in association with an increase in velocity in anaerobic threshold during treadmill exercise. Good aerobic fitness is beneficial for cardiovascular autonomic function [17]. The increase in heart rate during submaximal exercise is also related to lower maximal oxygen uptake [18]. Autonomic modulation induced by acupuncture stimulation before exercise may improve exercise performance. In addition, Toma et al. [19] reported that acupuncture stimulation attenuates the increase in skin sympathetic nerve activity elicited by handgrip exercise. They also observed that this suppression of exercise-induced increase in skin sympathetic nerve activity was maintained throughout the handgrip exercise. Therefore, acupuncture stimulation may suppress exercise-induced tachycardia through alteration of autonomic nervous activity.

The lower heart rate response observed during low-intensity exercise and at the beginning of high-intensity exercise in this study suggests that the efficiency of myocardial oxygen utilization is increased during exercise after pre-exercise acupuncture. Improvement of aerobic fitness leads to a lower heart rate response during submaximal exercise [20] and the heart rate response is strongly correlated with myocardial oxygen consumption [8, 9]. Jorgensen et al. [8] stated that the heart rate is a good index of the metabolic demands of the heart during normal exercise. Therefore, the modulation of autonomic nervous activity by pre-exercise acupuncture may be attributable to altered myocardial metabolism leading to more efficient energy utilization during exercise.

On the other hand, the effects of acupuncture stimulation on heart rate response during exercise disappeared when post-acupuncture high-intensity exercise was performed at a heart rate of approximately 140 beats/min. The relative influence of the sympathetic and parasympathetic branches of the autonomic nervous system changes during resting conditions (acupuncture stimulation) and exercise. First, acupuncture stimulation at rest induces parasympathetic dominant influence. Then, as exercise workload increases, there is a gradual shift to greater sympathetic influence at a higher workload [7]. This suggests that the effects of pre-exercise acupuncture stimulation on the heart rate response during exercise depend on the sympathetic/parasympathetic (vagal) balance induced by the intensity of exercise.

However, Li et al. [21] noted that although 30 min of electrical acupuncture at rest did not alter blood pressure, the electroacupuncture reduced systolic blood pressure and mean blood pressure during an incremental exercise test. They also found that the exercise-related heart rate response was not affected by pre-exercise electroacupuncture stimulation. Karvelas et al. [22] demonstrated that 10 min of acupuncture stimulation at six acupoints, Baihui (WHO; Gv20), Juiwei (WHO; Co15), Zhongmen (WHO; Li13), Sanyinjiao (WHO; SP6), Neiguan (WHO; PC6), and Zusanli (WHO; ST36), had no effect on physiological responses, including oxygen uptake and heart rate, during submaximal exercise after acupuncture. In addition, Middlekauff et al. [23] reported that acupuncture did not attenuate heart rate or blood pressure responses during hand grip exercise and the cold pressure test. Different acupuncture points, stimulation duration, and stimulation intensity used in different studies may explain the discrepant results. Middlekauff et al. [23] stimulated the inserted acupuncture needles manually for approximately 15 s and then left the needles in place for 15 min. The duration of acupuncture stimulation used in our study was 10 min. Yamamoto et al. [14] reported that the stretch-activated channel blocker gadolinium significantly attenuated the acupuncture-induced bradycardic response, suggesting that mechanoreceptors play an important role in the sensory mechanism of both manual and electrical acupuncture. The insertion depth of the needle may influence the magnitude of cardiovascular responses to acupuncture. Acupuncture stimulation of the skin or muscle alone may also produce different effects on cardiovascular responses [13, 24]. Previous studies demonstrated that acupuncture stimulation applied to the muscle alone produced depressor [24] and bradycardic [13] responses, whereas that of skin alone did not induce these responses. Moreover, the heart rate response monitored within seconds and minutes may be a factor related to the different findings in studies. The cardiovascular responses elicited by acupuncture stimulation are reflex-mediated via somatic afferent nerves and autonomic efferent nerves [13, 15]. Therefore, the effects of acupuncture stimulation may strongly affect the heart rate response within a few seconds.

RPE is one of the most common methods of assessing internal load during exercise [25]. A previous study noted the relationship between RPE and physiological measurements, including heart rate, during exercise [26]. Stephen and Trenske [27] reported that RPE was influenced by physiological and psychological factors. RPE was also significantly lower under music conditions, which attenuated the feeling of discomfort during low-intensity exercise, but was not different between music and sensory deprived conditions during both moderate and high-intensity exercises. In our study, RPE was affected by acupuncture stimulation during low-intensity exercise, but not during high-intensity exercise. This suggests that acupuncture stimulation before exercise effectively influences physiological and psychological loads during low-intensity exercise.

## Possible implications

The acupuncture-induced heart rate-reducing effects remained during low-intensity exercise and at the beginning of high-intensity exercise. As mentioned above, the modulation of autonomic nervous activity elicited by pre-exercise acupuncture may be attributable to more efficient myocardial oxygen utilization during exercise. Moreover, the alteration in the subjective feeling of fatigue during exercise elicited by pre-exercise acupuncture stimulation was observed during low-intensity exercise, suggesting that pre-exercise acupuncture stimulation may be beneficial for exercise-induced feeling states during exercise. This study provides new insight into the effects of acupuncture stimulation on psycho-physiological conditions and performance during exercise.

## Study limitations

Several limitations of the study should be mentioned. First, blood pressure was not measured in this study. Previous human and animal studies reported that acupuncture stimulation elicits a depressor response [2, 4]. Kitamura et al.[9] also reported that heart rate and aortic blood pressure strongly correlated best with myocardial oxygen consumption, but the heart rate alone correlated almost as well. Further studies are needed to examine whether the pre-exercise acupuncture stimulation affects other cardiovascular parameters associated with exercise performance. Second, our study only evaluated the effects of pre-acupuncture stimulation on heart rate response during short-duration exercise (1 min) at two exercise intensities. Further studies are thus needed to assess the effects of pre-exercise acupuncture stimulation on heart rate responses during long-duration exercise and at other exercise intensities.

## Conclusions

Acupuncture stimulation induced a bradycardic response at rest, and the acupuncture-induced heart rate-reducing effects remained during low-intensity exercise and in the beginning of high-intensity exercise performed immediately after the cessation of acupuncture stimulation. The effects disappeared when post-acupuncture exercise was performed when the heart rate was approximately 140 beats/min during high-intensity exercise. This study may provide valuable information on the effects of acupuncture stimulation on exercise performance and the factors involved.

## Data Availability

The datasets generated during and/or analyzed during the current study are available from the corresponding authors upon reasonable request.

## References

[1] Nakahara H, Kawada T, Ueda SY, Kawai E, Yamamoto H, Sugimachi M, Miyamoto T (2016). Electroacupuncture most effectively elicits depressor and bradycardic responses at 1 Hz in humans. Clin Auton Res.

[2] Nakahara H, Kawada T, Ueda SY, Kawai E, Yamamoto H, Sugimachi M, Miyamoto T (2019). Acupoint dependence of depressor and bradycardic responses elicited by manual acupuncture stimulation in humans. J Physiol Sci.

[3] Nishijo K, Mori H, Yosikawa K, Yazawa K (1997). Decreased heart rate by acupuncture stimulation in humans via facilitation of cardiac vagal activity and suppression of cardiac sympathetic nerve. Neurosci Lett.

[4] Michikami D, Kamiya A, Kawada T, Inagaki M, Shishido T, Yamamoto K, Ariumi H, Iwase S, Sugenoya J, Sunagawa K, Sugimachi M (2006). Short-term electroacupuncture at Zusanli resets the arterial baroreflex neural arc toward lower sympathetic nerve activity. Am J Physiol Heart Circ Physiol.

[5] Gentil D, Assumpção J, Yamamura Y, Barros Neto T (2005). The effect of acupuncture and moxibustion on physical performance by sedentary subjects submitted to ergospirometric test on the treadmill. J Sports Med Phys Fitness.

[6] Lin ZP, Lan LW, He TY, Lin SP, Lin JG, Jang TR, Ho TJ (2009). Effects of acupuncture stimulation on recovery ability of male elite basketball athletes. Am J Chin Med.

[7] White DW, Raven PB (2014). Autonomic neural control of heart rate during dynamic exercise: revisited. J Physiol.

[8] Jorgensen CR, Wang K, Wang Y, Gobel FL, Nelson RR, Taylor H (1973). Effect of propranolol on myocardial oxygen consumption and its hemodynamic correlates during upright exercise. Circulation.

[9] Kitamura K, Jorgensen CR, Gobel FL, Taylor HL, Wang Y (1972). Hemodynamic correlates of myocardial oxygen consumption during upright exercise. J Appl Physiol.

[10] Zhou W, Fu LW, Tjen-A-Looi SC, Li P, Longhurst JC (2005). Afferent mechanisms underlying stimulation modality-related modulation of acupuncture-related cardiovascular responses. J Appl Physiol (1985).

[11] Gabriel H, Urhausen A, Kindermann W (1992). Mobilization of circulating leucocyte and lymphocyte subpopulations during and after short, anaerobic exercise. Eur J Appl Physiol Occup Physiol.

[12] de Souza DC, Matos VAF, Dos Santos VOA, Medeiros IF, Marinho CSR, Nascimento PRP, Dorneles GP, Peres A, Müller CH, Krause M, Costa EC, Fayh APT (2018). Effects of High-Intensity Interval and Moderate-Intensity Continuous Exercise on Inflammatory, Leptin, IgA, and Lipid Peroxidation Responses in Obese Males. Front Physiol.

[13] Uchida S, Kagitani F, Hotta H (2010). Neural mechanisms of reflex inhibition of heart rate elicited by acupuncture-like stimulation in anesthetized rats. Auton Neurosci.

[14] Yamamoto H, Kawada T, Kamiya A, Miyazaki S, Sugimachi M (2011). Involvement of the mechanoreceptors in the sensory mechanisms of manual and electrical acupuncture. Auton Neurosci.

[15] Uchida S, Shimura M, Ohsawa H, Suzuki A (2007). Neural mechanism of bradycardiac responses elicited by acupuncture-like stimulation to a hind limb in anesthetized rats. J Physiol Sci.

[16] Cheung LC, Jones AY (2007). Effect of Acu-TENS on recovery heart rate after treadmill running exercise in subjects with normal health. Complement Ther Med.

[17] Tulppo MP, Mäkikallio TH, Seppänen T, Laukkanen RT, Huikuri HV (1998). Vagal modulation of heart rate during exercise: effects of age and physical fitness. Am J Physiol.

[18] Arngrímsson SA, Stewart DJ, Borrani F, Skinner KA, Cureton KJ (2003). Relation of heart rate to percent VO2 peak during submaximal exercise in the heat. J Appl Physiol.

[19] Toma K, Walkowski S, Metzler-Wilson K, Wilson TE (2011). Acupuncture attenuates exercise-induced increases in skin sympathetic nerve activity. Auton Neurosci.

[20] Carter JB, Banister EW, Blaber AP (2003). Effect of endurance exercise on autonomic control of heart rate. Sports Med.

[21] Li P, Ayannusi O, Reid C, Longhurst JC (2004). Inhibitory effect of electroacupuncture (EA) on the pressor response induced by exercise stress. Clin Auton Res.

[22] Karvelas BR, Hoffman MD, Zeni AI (1996). Acute effects of acupuncture on physiological and psychological responses to cycle ergometry. Arch Phys Med Rehabil.

[23] Middlekauff HR, Shah JB, Yu JL, Hui K (2004). Acupuncture effects on autonomic responses to cold pressor and handgrip exercise in healthy humans. Clin Auton Res.

[24] Ohsawa H, Okada K, Nishijo K, Sato Y (1995). Neural mechanism of depressor responses of arterial pressure elicited by acupuncture-like stimulation to a hindlimb in anesthetized rats. J Auton Nerv Syst.

[25] Halson SL (2014). Monitoring training load to understand fatigue in athletes. Sports Med.

[26] Scherr J, Wolfarth B, Christle JW, Pressler A, Wagenpfeil S, Halle M (2013). Associations between Borg's rating of perceived exertion and physiological measures of exercise intensity. Eur J Appl Physiol.

[27] Stephen B, Trenske M (1990). The effects of sensory deprivation and music on perceived exertion and affect during exercise. J Sport Exerc Psychol.

